# Leveraging the ROS–TME Axis for Cancer Treatment

**DOI:** 10.3390/antiox13111365

**Published:** 2024-11-07

**Authors:** Kostas A. Papavassiliou, Amalia A. Sofianidi, Vassiliki A. Gogou, Athanasios G. Papavassiliou

**Affiliations:** 1 First University Department of Respiratory Medicine, ‘Sotiria’ Chest Hospital, Medical School, National and Kapodistrian University of Athens, 11527 Athens, Greece; konpapav@med.uoa.gr (K.A.P.); gogvanessa@gmail.com (V.A.G.); 2 Department of Biological Chemistry, Medical School, National and Kapodistrian University of Athens, 11527 Athens, Greece; amsof.00@gmail.com

The discovery of reactive oxygen species (ROS) dates back to the early 20th century [1], yet their role in essential biological processes has only recently begun to near being fully understood. ROS are a versatile group of highly reactive chemical molecules derived from oxygen and produced during normal cellular metabolism [2]. This group includes non-radical species like hydrogen peroxide (H_2_O_2_) and free radicals like superoxide (O_2_^•−^) alongside other oxidants [2]. ROS play a vital role as signaling molecules in critical biological processes like cell proliferation, differentiation, apoptosis, and immune responses [2]. Notably, the key to preserving these functions is maintaining low levels of ROS inside the human body; redox homeostasis—a balanced state where ROS levels are tightly regulated—helps to prevent oxidative stress and protects cells from its potentially detrimental effects [3].

In cancer, redox homeostasis is often disrupted, leading to elevated oxidative stress within cancer cells. While it may seem that oxidative stress would harm cancer cells the same way it harms normal cells, the reality is quite different: moderate levels of ROS are beneficial to cancer cells, promoting proliferation, angiogenesis, survival, and metastasis [4,5]. ROS production has been linked to increased levels of the transcription factor nuclear factor-kappa B (NF-κB), which is recognized for its role in fostering tumorigenesis and inducing epithelial–mesenchymal transition (EMT), a process that facilitates distant metastasis [6]. However, because excessive amounts of ROS beyond a certain level can be lethal, cancer cells have developed enhanced antioxidant capacities [7]. Within the tumor microenvironment (TME), ROS levels stimulate the entry of the transcription factor nuclear factor erythroid 2-related factor 2 (NRF2) into the nucleus, potentiating the expression of genes responsible for the synthesis of glutathione (GSH) [7]. This antioxidant response helps to alleviate oxidative stress in cancer cells, supporting their continued growth and resilience [7] and contributing to a chemoresistant phenotype [8].

On the other hand, in the TME, cancer cells and immunosuppressive cells exploit ROS to foster immune tolerance towards tumors [9]. ROS has been found to upregulate the expression of the inhibitory molecule programmed death-ligand 1 (PD-L1) as well as the stimulatory molecule cluster of differentiation 80 (CD80) on antigen-presenting cells (APCs), influencing the dynamic interaction between these cells and T cells [9]. Additionally, ROS triggers hypoxia, suppressing the activation of T cells, natural killer (NK) cells, and dendritic cells (DCs) [9]. Pathologically high levels of ROS in the TME further shape immune responses by promoting the transition of monocytes into M2 macrophages while reducing M1 macrophages, thereby augmenting an immunosuppressive state [9]. Notably, high levels of ROS have been linked to poor response rates and insensitivity to immunotherapy [10].

Given the link between ROS and the TME, targeting the ROS–TME axis has become a promising focus in cancer treatment research. Maintaining their distinct redox homeostasis is essential for the survival of cancer cells, cancer stem cells, cancer-associated fibroblasts (CAFs), and other cells within the TME. Shifting the balance between ROS production and elimination in either direction can profoundly affect cancer cell survival and drug sensitivity. To harness the ROS pathway for cancer therapy, two key strategies have been proposed: targeting either ROS production or ROS scavenging mechanisms [11]. To target the ROS generation machinery in cancer cells, the inhibition of nicotinamide adenine dinucleotide phosphate (NADPH) oxidase (NOX) is considered to be a promising strategy [11]. Since CD44 is aberrantly expressed in cancer cells regulating metastasis via the recruitment of CD44 to the cell surface, CD44-targeted nanoparticle systems to deliver NOX inhibitors have been developed and proven to be effective both in vitro and in vivo [11]. Notably, it appears that knocking down NOX4 with small interfering RNA (siRNA) or blocking its activity using setanaxib (a first-in-class NOX1/4 dual inhibitor) can help to overcome resistance to immunotherapy [12]. Inhibition of nitric oxide synthase (NOS) has also been proposed in preclinical models in an effort to reduce the production of ROS that is beneficial for malignant cells [11]. Conversely, enhancing cytotoxic ROS production offers an intriguing approach by intensifying oxidative stress to selectively damage cancer cells [11]. Preclinical models revealed favorable antitumor activity by upregulating ROS-generating enzymes such as NOX5, achieved through the use of anlotinib, a small-molecule multitarget tyrosine kinase inhibitor (TKI) [13]. Moreover, abolishing the metabolic enzyme mitochondrial isocitrate dehydrogenase 2 (IDH2) has been shown to promote ROS production in vitro, while enhancing the sensitivity of lung cancer cells to cisplatin in vivo [14].

The ROS scavenging mechanisms of cancer cells include the activation of the antioxidant molecules TP53 inducible glycolysis and apoptosis regulator (TIGAR; a bisphosphatase that attenuates glycolysis) and NRF2. Interestingly, targeting these antioxidant molecules seems to have a paradoxical effect: while tumor initiation is delayed, the potential for metastasis is enhanced [15,16]. Nevertheless, antioxidant treatment with N-acetyl-L-cysteine (NAC) reduced metastasis in mice with TIGAR-knockout pancreatic tumors [15], which is in contrast with the promotion of metastasis in melanoma [17] and lung cancer preclinical models [18]. Hindering GSH activity is another promising therapeutic strategy; blocking GSH with β-phenylethyl isothiocyanate (PEITC), a natural GSH-conjugating photochemical, can induce ROS accumulation and cancer cell death [11,19]. Blocking GSH peroxidases (GPXs) or thioredoxin reductases (TrxRs), enzymes responsible for ROS neutralization, has also demonstrated preclinical effectiveness [11].

The use of pro-oxidants has also been extensively studied for their impact on ROS levels and effects on cancer cells. Certain pro-oxidants have been found to enhance the efficacy of cancer immunotherapy [5]. Among the promising candidates are pharmacological doses of ascorbate, nonsteroidal anti-inflammatory drugs (NSAIDs), cyst(e)inase, inhibitors of the cystine-glutamate antiporter SLC7A11/xCT, and ROS-responsive prodrugs that activate cytotoxicity specifically in the presence of ROS and are delivered via nanoparticles [5]. Notably, ascorbate and avasopasem manganese—a mimetic of the enzyme superoxide dismutase (SOD)—have been found to act as pro-oxidants in cancer cells and antioxidants in healthy cells, but the underlying mechanisms are not yet elucidated [11,20,21,22].

In summary, the therapeutic potential of targeting the ROS–TME axis has been explored only in preclinical models so far. What remains to be determined is whether these observations will translate effectively to clinical settings. Novel agents that target ROS in the TME could focus on CAFs and their interplay with malignant cells [11]. Remarkably, the equilibrium between ROS production and ROS scavenging is extremely delicate in cancer cells. Disrupting this balance either by generating high oxidative stress or by eliminating ROS has the potential to hamper key hallmarks of cancer, offering promising therapeutic opportunities in the cancer field.
